# A data-driven approach to improve wellness and reduce recurrence in cancer survivors

**DOI:** 10.3389/fonc.2024.1397008

**Published:** 2024-04-11

**Authors:** Ramkumar Hariharan, Leroy Hood, Nathan D. Price

**Affiliations:** ^1^ College of Engineering, Northeastern University, Seattle, WA, United States; ^2^ Institute for Experiential Artificial Intelligence, Northeastern University, Boston, MA, United States; ^3^ Institute for Systems Biology, Seattle, WA, United States; ^4^ Buck Institute for Research on Aging, Novato, CA, United States; ^5^ Phenome Health, Seattle, WA, United States; ^6^ Thorne HealthTech, New York, NY, United States

**Keywords:** cancer survivor, scientific wellness, long-term effects, late effects, quality of life, dense dynamic personal data, healthspan, cancer recurrence prevention

## Abstract

For many cancer survivors, toxic side effects of treatment, lingering effects of the aftermath of disease and cancer recurrence adversely affect quality of life (QoL) and reduce healthspan. Data−driven approaches for quantifying and improving wellness in healthy individuals hold great promise for improving the lives of cancer survivors. The data-driven strategy will also guide personalized nutrition and exercise recommendations that may help prevent cancer recurrence and secondary malignancies in survivors.

## Introduction: QoL issues in survivors living with cancer and reduced healthspan in cancer–free survivors

Due to advances in early diagnosis and treatment, together with a population with an increasing proportion of older people, the number of cancer survivors in the US is expected to reach 22.1 million by 2030 (1, http://cancerstatisticscenter.cancer.org. Accessed Dec 29, 2023). Most cancer survivors face varying degrees of compromised quality of life (QoL) issues, resulting in reduced healthspan even for those for whom primary treatment was successful and have become cancer-free (2). QoL issues stem from either damage inflicted by the cancer itself, from toxic side effects of therapy (ies) or from a combination of both. While early cancer detection and treatment have improved in recent years, efforts to regain wellness and enhance healthspan in survivors has lagged behind (3). In both survivors living with cancer and in individuals who are cancer-free, optimal wellness goals should include ensuring maximal QoL while preventing cancer recurrence or halting cancer progression. Indeed, it may prove that such goals are well aligned as increasing overall health may help increase resilience to recurrence. As a case in point, in 2022, the American Cancer Society (ACS), after ten years, updated their dietary and exercise recommendations for survivors. At least for survivors of prostate, colorectal and breast cancers, exercise and optimal nutrition appears to be associated with a lower risk of disease relapse and mortality (4–7). Thus, there’s a pressing need to quantitatively transform these observations into personalized lifestyle recommendations for survivors with a view towards especially dealing with cancer complications.

Long-term and late effects are two common types of side effects (8). Long-term effects typically start *during* cancer treatment and *linger on* for weeks to months after termination of therapy (8). In contrast, late effects show up only *after* termination of cancer therapy (2, 9). For example, in some pediatric cancer survivors, later-effects of treatment have manifested several decades after treatment (3) and include cardiac problems.

Here, we briefly discuss some of the more common side-effects of standard cancer therapies before discussing scientific wellness strategies (10, 11) to ameliorating them, reducing risk or early detection of cancer recurrence in survivors.

## Long-term and late effects of cancer treatment

### Cancer-related cognitive impairment

Of survivors with non-central nervous system cancers treated with chemotherapy, around 40-75% suffer from so-called ‘chemobrain’ (i.e. ‘brain fog’) (12, 13). Chemobrain refers to problems with short term memory, inability to focus on a task, impaired information processing, and issues with executive function (12). Such mild to moderate cognitive impairment following chemotherapy, is typically a long-term side effect that lasts for a few months or even years after end of treatment. However, it can sometimes show up as a late effect as well (12).

Several pathophysiological correlates of chemobrain have emerged from recent studies. Some of the more proximal neural correlates with chemobrain come from imaging studies involving cancer survivors. For example, brain imaging studies in cancer survivors have identified a diffuse reduction in white matter and grey matter volume following chemotherapy (14). White matter microstructure also seems to be affected by some chemotherapeutic regimens (14). Other observations in chemotherapy-treated patients include altered activation patterns of cortical networks (15, 16), and changes in brain glucose metabolism that underlie frontal hypometabolism (17). Distal mechanisms have been less well elucidated in chemobrain patients, but studies in mice have shown that commonly used chemotherapeutic regimens can reduce cell proliferation and alter histone modification in hippocampal neural progenitor cells (18). These changes contribute to impaired hippocampal neurogenesis, which can lead to cognitive dysfunction (19, 20). Other studies report that chemotherapy can enhance protein oxidation which in turn activates neurotoxic pro-inflammatory cascades (18, 21, 22). Additionally, genetic polymorphisms in several genes, including those that encode *BDNF* and *APOE4*, have been shown to modulate severity of chemobrain (23, 24).

Chemobrain can significantly disrupt an individual’s professional, personal, and social life. There is clearly a need to prospectively identify early and treat cancer survivors who are at high risk of developing chemobrain after therapy. For such high-risk individuals, we need early interventions employed concomitant with initiation of chemotherapy to effectively prevent or at least mitigate chemobrain. Abnormal circulating levels of pro-inflammatory cytokines including Tumor Necrosis Factor-α (TNF-α), its soluble receptor and a few other interleukins have also been correlated to chemobrain in cancer survivors (21). However, reliable clinical biomarkers for chemobrain do not yet exist, and there is not yet a deep understanding of the mechanisms on which to best intervene.

### Chemotherapy-induced arthralgia

Post-menopausal women treated with aromatase inhibitors can develop arthralgia ─ pain and stiffness in their joints, as a long-term side effect of therapy (25). The severity and persistence of arthralgia in these patients is a major factor that drives treatment non-adherence, and in turn leads to shortened survival (26). Exercise regimens and treatment with non-steroidal anti-inflammatory agents (NSAIDS) for managing CIA are typically used in the clinic but with limited effectiveness (25). A possible etiology for CIA is reduced estrogen level triggered by aromatase inhibitor therapy (27). However, a more comprehensive understanding of the mechanisms that underlie CIA are needed to develop effective interventions.

### Chemotherapy-induced peripheral neuropathy

Another long-term effect of multiple chemotherapy regimens that contribute to treatment non-adherence is CIPN (26, 28). Onset of CIPN in survivors seems to be dose-dependent; a minority of patients will experience CIPN at moderate chemotherapy doses, but most will only have CIPN at high doses (28).

The pathophysiology of CIPN is believed to be distinct from that of other kinds of peripheral neuropathy (29). Some of the proposed causal mechanisms include chemotherapy-induced mitochondrial dysfunction that triggers apoptosis, altered levels of pain mediators, and aberrant spontaneous activity in A and C fibers (29). While there is no consensus on standard-of-care for CIPN in cancer survivors, a number of medications have shown some benefit in these individuals (28).

### Fatigue

Fatigue is a long-term effect and can persist for years after termination of cancer therapy. It reduces QoL and healthspan in survivors and compounds the severity of other side effects (30). Pathophysiological underpinnings of fatigue in cancer survivors include elevated pro-inflammatory activity and insomnia (31, 32).

### Obesity and nutrition

Obesity and sub-optimal nutrition in cancer survivors not only influence QoL and healthspan, but also negatively modify prognosis and treatment outcomes (33). Obesity can feed cancer progression and adversely modify some of the other side effects of cancer treatment through multiple mechanisms (33). New pharmaceuticals developed for diabetes treatment such as tirzepatide and semaglutide have recently shown remarkable ability to reduce weight in obese patients without diabetes (34, 35). It remains to be tested how well such interventions might be tolerated in cancer survivors and what the benefit and side effect trade-offs of such treatments might be. Muscle loss and increases in Thyroid cancers are amongst the concerns. Physical activity and nutrition guidelines have been proposed to address obesity and malnutrition in cancer survivors (36). However, since energy balance, metabolism, and nutritional needs for each survivor is different, a personalized, quantitative and continuous approach to address this problem is warranted.

### Stress and anxiety

High levels of stress and anxiety are prevalent among cancer survivors, and adversely affect QoL and healthspan (37). The few stress-reduction programs employed in the clinic to date have met with varying degrees of effectiveness (38). As with nutrition, a continuous, personalized approach to monitoring and managing stress in cancer survivors is needed.

### Cardiovascular disease and metabolic syndrome

CVD develops as a serious late effect in between 4 to 30% of long-term survivors of childhood and adult malignancies treated with anthracyclines, certain targeted therapies, and radiotherapy (39, 40). Patients treated with anthracyclines are at risk for developing congestive heart failure due to drug-induced cardiomyopathy (41). Cardiac arrhythmias and myocarditis constitute other treatment-related cardiotoxicities (42).

CVD risk in cancer survivors is compounded by metabolic syndrome, which arises from a cluster of factors including dyslipidemia, hypertension, and insulin resistance. Multiple studies have found a higher prevalence of metabolic syndrome in long-term cancer survivors as compared to the general population (43). It has also been suggested that metabolic syndrome in cancer survivors may have a different pathophysiology from that observed in the general population. An altered hormonal axis triggered by radiation- or chemotherapy might partly explain a cancer survivors’ increased risk of developing CVD (43).

The presence of specific genetic variants, female sex, and other pre-existing co-morbidities are all risk factors that can predispose cancer patients to developing cancer treatment-induced cardiotoxicities (40). However, biomarkers to prospectively identify cancer patients at high risk of developing treatment-induced cardiotoxicities do not exist (44, 45). The etiology of metabolic syndrome in cancer survivors also needs to be understood in more detail to enable the development of better, more rational interventions.

### Secondary malignancies

Treatment-related secondary malignancies are a rare but serious late-effect of certain cancer therapeutic regimens (46, 47). Prospectively identifying survivors at high-risk for developing treatment–associated second cancers can prioritize them for early screening.

### Other long-term effects

Lymphedema after surgery (48), and chemotherapy-induced persistent diarrhea are other long-term effects in survivors (49). The role of gut microbiota in modulating irinotecan (an anticancer drug) induced diarrhea has been documented (50). A recent study found that chemotherapy can bring about severe compositional changes in the gut microbiome, in turn triggering intestinal dysbiosis, which eventually leads to gastro-intestinal mucositis (51, 52). The role of the intestinal microbiome in modulating toxic effects of cancer therapy needs to be further explored.

### Cancer recurrence

Chances of cancer recurrence depend on various cancer, patient, and treatment related factors including the grade, stage of tumor at diagnosis and, the type of cancer. Recurrence rates range from a low 5% for estrogen positive breast cancers (53) to nearly 100% for glioblastoma multiforme (54), with other tumor types falling somewhere in between. Except for a handful of cancer types, including prostate, chronic myeloid leukemia (CML) and colorectal cancer, useful markers of cancer recurrence do not exist. Dietary and exercise recommendations for cancer prevention often prevent disease recurrence as well. Again, wellness biomarkers that vary longitudinally could serve not only as early biomarkers of recurrence but guides to personalized diet and physical activity recommendations to prevent the complications. Cancer survivors free of disease are excellent candidates to study longitudinally to identify blood biomarkers for early disease reoccurrence. Detecting early re-emergences of cancers when they are simple in their disease complexity, offers an excellent opportunity to explore effective preventive treatments.

## Personal, dense, dynamic data clouds as a path to wellness in survivors

Given the myriad issues detailed above for cancer survivors, and their growing numbers, better, and more global, solutions are needed than just attempting to treat individual symptoms. Many of these can be achieved by leveraging data-driven approaches to enhancing health. The feasibility and utility of gathering longitudinal, multi-tiered data from individuals has been demonstrated in recent studies (10, 55–57) and focused on the development of what we call scientific wellness (58).

In 2014, the first of these cohort studies, the Pioneer 100 Wellness Project (P100) was carried out at the Institute for Systems Biology. The P100 generated personal, dense, dynamic data clouds from a cohort of 108 individuals, sampled on a quarterly basis over 9 months (10). Each participant had their whole genome sequenced as well as longitudinal sampling at 3-month intervals of a large battery of over 100 clinical blood tests, metabolomics and proteomics from the blood, and microbiome from the stool. Additionally, Fitbits were used to track daily activity and tracking of goals and results was monitored and facilitated individually with a health coach. The authors identified thousands of molecular correlates across the various datatypes, including effects of genetics on the metabolome and proteome. Further, when these data were used to guide highly personalized behavioral coaching, participants improved their clinical biomarkers of health and wellness (10).

From 2015-2019, a similar ‘scientific wellness’ program was offered by Arivale (co-founded by L.H. and N.D.P) that generated longitudinal multi-omic datasets on approximately 5000 people who gave consent for their de-identified data to be used for research purposes. Similar to the P100 study, the program participants, on average, achieved sustained, significant improvements in clinical markers of cardiometabolic risk, inflammation, nutrition, and body mass index (BMI). Improvements in HbA1c levels matched those observed in other landmark clinical trials (59). By finding genetic markers associated with longitudinal changes in such clinical markers, the study also concluded that genetic predisposition impacts clinical responses to lifestyle change in distinct manners for individuals of high and low genetic risk (60).

Subsequent studies using these identified 766 multi-omic associations in the blood related to polygenic risk scores for 54 diseases and traits to provide clues about prevention strategies for prodromal disease (61), discovered early candidate protein biomarkers of cancer and cancer metastasis (62), proposed multi-omic models of biological age as a measure of wellness (55), identified microbiome-derived candidate biomarkers of cardiovascular disease (63) and revealed a key role for the microbiome in healthy aging (Wilmanski et al). Specifically, the Magis et al. study identified carcinoembryonic antigen-related cell adhesion molecule 5 (CAECAM 5), as a persistent, longitudinal outlier, showing up as early as 26 months before individuals were diagnosed with breast, lung or pancreatic cancer. CAECAM 5 is known to be overexpressed in the primary versions of these three cancers and also in metastatic disease. In this case, the scientific wellness data enabled the discovery that such biomarkers of cancer metastasis rose well before diagnosis. Additionally, the study found two additional persistent outliers – calcitonin-related polypeptide alpha (CALCA) and delta-like 1 homolog (DLK1), for metastatic pancreatic cancer. All three proteins would serve as candidate early biomarkers for metastatic disease.

Similar datasets in cancer survivors could provide critical insights to improving health in these populations as well. Actionable discoveries from the scientific wellness approach offer promise to shape choices of intervention for reducing toxic side-effects. This is especially relevant since radiation therapy, hormone therapy and chemotherapy are all known to accelerate biological age in patients (Jacob 64). Their success can be measured using a range of metrics, including objective and subjective measures of QoL, biological age estimates (physiology-based, mutli-omic) and established measures of cognition and wellness. In addition, in cancer survivors, longitudinal phenomic analyses will be useful in detecting reoccurrences early.

Another recent study (56) used integrative omics and data from wearable monitoring to demonstrate the usefulness of longitudinal molecular and physiological deep profiling in precision health. The study authors assayed the genome, transcriptome, proteome, metabolome, immunome and the microbiome, all paired with quarterly enhanced clinical tests, of 109 individuals up to 8 years. These individuals were at elevated risk for developing type II diabetes. Importantly, they were able to discover “molecular pathways associated with metabolic, cardiovascular and oncological pathophysiologies”. Additionally, they made 67 clinically actionable discoveries, developed an omics-based model for insulin resistance and fomented healthy dietary and exercise behaviors in the study participants.

The feasibility and stability of building longitudinal, personalized omics profiles of wellness was demonstrated by another recent study in which 100 healthy subjects not only showed the surprising stability of blood plasma protein profiles over time but also their significant association with blood chemistry (57). For example, the authors found a strong association between C- Reactive Protein (synthesized by the liver and a biomarker of inflammation) and Interleukin – 6 (an immune system cytokine related to inflammation). In agreement with other recent studies, the intra-individual baseline variability of omics profiles was low compared to the inter-individual ones.

Thus, longitudinal, high-throughput multi-omic data generation and analysis can be used to (1) define omics baselines of wellness, (2) define early wellness-to-disease and disease-to-wellness transitional omics signatures, (3) discover molecular pathways that underlie specific pathology, (4) find potential biomarkers of disease and (5) help guide and optimize personalized wellness coaching for individuals.

Cancer survivors stand to benefit from such a scientific wellness approach for several reasons as discussed below. Survivors make up a specific group with limited traits, which improves the ability to find meaningful information in a long-term study of large data sets, even with relatively small sample size.

First and foremost, the longitudinal, multi-tiered data collection will enable us to build an integrated baseline in this population. Deviations from this baseline can inform the discovery of novel diagnostic and prognostic biomarkers of various toxic side-effects of therapy discussed above and equally importantly, those of cancer recurrence. For example, data from survivors can be mined to potentially discover new temporal signatures (consisting of metabolites, peptides, proteins or microbiota) that correlate with known prognostic or diagnostic biomarkers (e.g. PSA and BCR ABL, respectively, for prostate and CML recurrence) (65, 66). The same approach can also be used in survivors to find novel, more effective biomarkers to monitor the effectiveness of maintenance therapy used in some cancer patients even after achieving complete remission.

Second, scientific wellness approaches can be a powerful tool to assess and eventually better tailor, diet and physical activity protocols for survivors with a goal of preventing cancer recurrence and accelerating their return to wellness. This is a corollary to the previously mentioned, new and revised guidelines from the NCI, with regard to diet and physical activity, for cancer survivors.

Third, the longitudinal multimodal data gathering, along with clinical records, will allow us to ask questions around correlative links between other comorbidities like obesity, diabetes or chronic inflammation and cancer recurrence. While the associations between conditions such as obesity and risk of cancer are known, data from our approach will provide a quantitative framework to better assess risks and make new biological discoveries.

Finally, the data clouds from survivors who go on to develop recurrences, could offer insights into tumor evolution from a new point of view (67) and yield new insights into transitions to a state of cancer recurrence. As was true with the other wellness studies, it appears that malignant lesions of the same cancer type, from the same patient are more alike than those from different patients (67).

**Figure 1:**
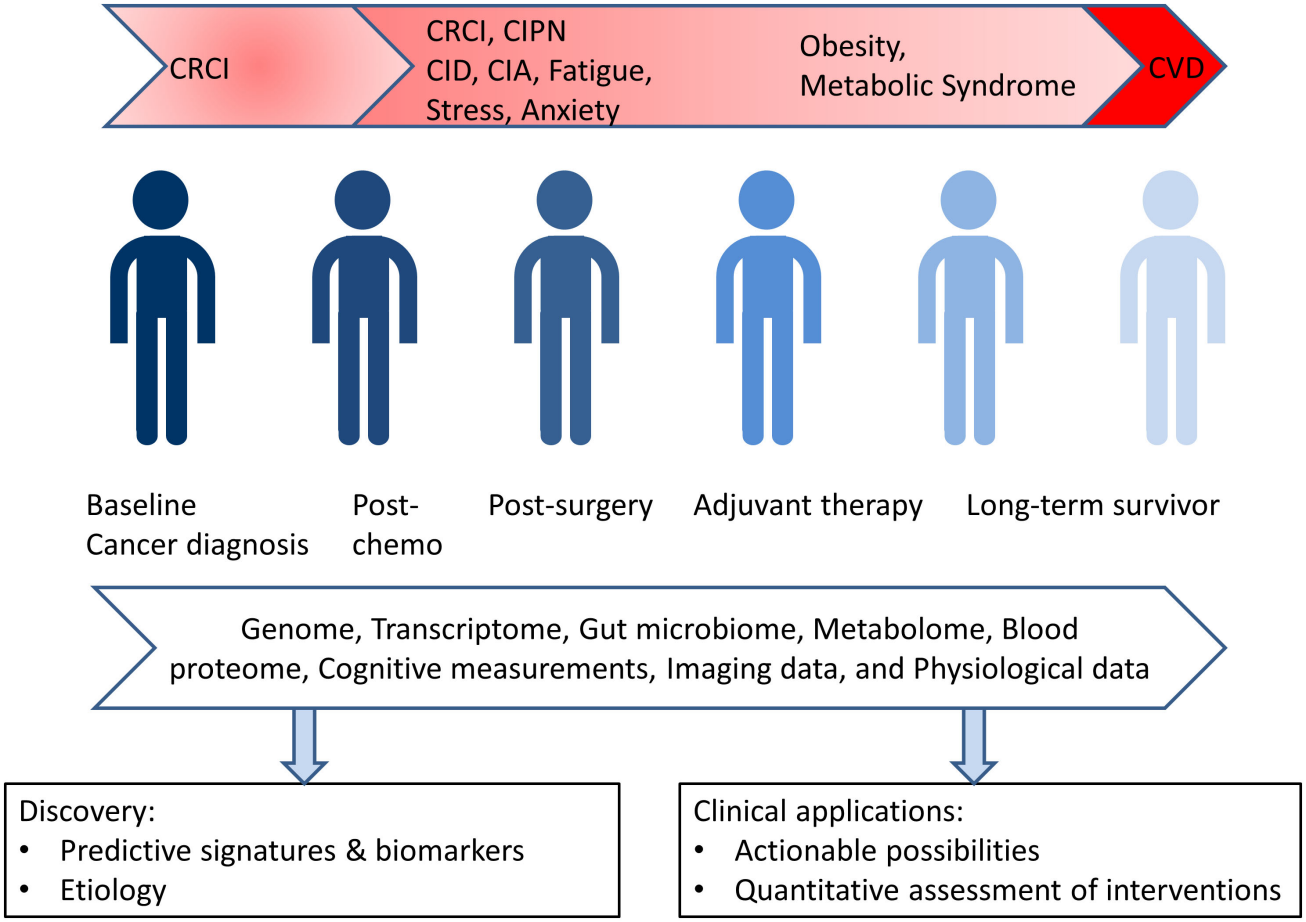
A dense, dynamic personalized data cloud path to wellness for cancer survivors.

A longitudinal, big data approach will provide a foundation for future studies in this area, and it will represent a first of its kind resource that can provide a foundation for future studies in this direction. This approach will generate virtual and dynamic clouds myriad data points that provide unique and personalized insights into the wellness and disease of each individual (see **Figure 1**). We are engaged in two such trials currently focused on cancer survivors: one is a collaboration between Mayo Clinic and Thorne HealthTech and the other is a collaboration between the Institute for Systems Biology and the Swedish Cancer Institute of Providence Health.

As a specific example, metabolomic data for each survivor along with relevant clinical lab data enables monitoring of integral components of metabolic syndrome. Using these dynamic data, a shift towards the metabolic state can be identified and may open up opportunities for early intervention. The data will also allow the effects of physical activity and other medical interventions on the various health metrics of the cancer survivor to be assessed. Other covariates from the data cloud can also be identified in the cohort of survivors, and these will open-up new avenues for intervention and exploration.

Another instance of the translational potential of this approach is the monitoring of pro- and anti-inflammatory cytokines in the blood of cancer survivors. Inflammation is thought to underlie many of the long-term and late-effects after cancer treatment including chemobrain, arthralgia, fatigue, and the metabolic syndrome (18, 25, 32, 43). Therefore, analyzing cytokine levels from each survivor across the cohort will enable discovery of prognostic and predictive biomarkers for these effects.

The longitudinal multi-omic protocol will complement existing, state-of-the-art tumor sequencing methods that provide predictive, prognostic and diagnostic value. Examples of these approaches include the Oncopanel by Eurofin and some other custom panels offered by some cancer centers and clinics in the US (Mayo clinic, MD Anderson and Memorial Sloan Kettering Cancer Center). The scientific wellness approach will bring additional value since it is longitudinal, multi-tiered (not just sequencing) and not tumor tissue specific. The combined value of enriching existing methods that offer a snapshot, with multi-parametric measurements spread over several time points can be evaluated in clinical trials.

Additionally, the dense, dynamic, personal data clouds will contain data from blood proteins which are emerging as potential biomarkers for organ function, especially the heart (45). Importantly, such biomarkers, taken together with physiological data from the same data clouds, will not only yield clues to discover underlying pathophysiological pathways, but will also identify time-points for early medical intervention. Finally, microbiome data from these data clouds can lead to discovery of new associations with disease, wellness, and ways to improve survivor health via the microbiome (68).

We anticipate that moving from carefully selected cohorts to a patient-centric personalized treatment plan will be a multi-step process, requiring carefully planning and evaluation all along the way. The number of studies and patients that would be required to arrive at a clinically translatable recommendation will depend on a variety of factors – robustness of biomarker validation, clinical trial outcomes and other regulatory considerations. The sample sizes provided by the initial exploratory studies provide clues on the effect sizes and can help estimation of the number of participants for future clinical trials.

## Concluding remarks

In summary, robust molecular correlates do not exist for most of the long-term and late effects of cancer treatment; the identification of molecular signatures and biomarkers underlying such effects will enable early interventions, and subsequently to their better management in the clinic. Beyond cancer diagnosis and treatment that are rightly the primary focus, further work is needed to improve wellness and quality of life in the increasing number of cancer survivors, including reduction in their co-morbidities and the early detection of transitions back to a cancerous state. The multi-parameter data from the cancer survivor cohort will allow the generation of sophisticated and highly accurate predictive models for such toxic side-effects and for cancer recurrence. These models can catalyze the discovery of new genetic modifiers for many of the treatment effects, predict high-risk sub-populations, and will help pave a more certain and accelerated path back to wellness for cancer survivors.

## Data availability statement

The original contributions presented in the study are included in the article/supplementary material. Further inquiries can be directed to the corresponding author.

## Author contributions

RH: Conceptualization, Writing – original draft. NP: Conceptualization, Supervision, Writing – review & editing. LH: Conceptualization, Writing – review & editing.
